# Fishing for protective compounds

**DOI:** 10.7554/eLife.61547

**Published:** 2020-09-03

**Authors:** Giuliano Ciarimboli

**Affiliations:** Experimental Nephrology, Med. Clinic D, Muenster University HospitalMuensterGermany

**Keywords:** cisplatin, drug screen, toxicity, nephrotoxicity, dopamine, L-mimosine, Human, Zebrafish

## Abstract

A new zebrafish study identifies compounds that shield ears and kidneys against an anticancer drug.

**Related research article** Wertman JN, Melong N, Stoyek MR, Piccolo O, Langley S, Orr B, Steele SL, Razaghi B, Berman JN. 2020. The identification of dual protective agents against cisplatin-induced oto-and nephrotoxicity using the zebrafish model. *eLife* **9**:e56235. doi: 10.7554/eLife.56235

Cancer treatments have become increasingly effective over the past few decades, but the chemotherapy drugs that kill tumour cells also damage healthy tissues. This can lead to serious side effects that go on to impair the quality of life of patients after recovery. For instance, cisplatin, a drug used to treat testicular cancer, is toxic to kidneys and hair cells in the ear that are necessary for hearing processes (Daugaard, 1990; Einhorn, 2002; Rybak and Ramkumar, 2007; Pabla and Dong, 2008; Lanvers-Kaminsky et al., 2017). Now, in eLife, Jason Berman and colleagues in institutions across Canada – including Jamie Wertman as first author – report the results of a study screening for compounds that reduce the toxicity of cisplatin (Wertman et al., 2020).

To do so, the team enlisted the zebrafish *Danio rerio*, a tiny freshwater tropical fish similar to humans at the molecular level, but can be bred cheaply and quickly (Schartl, 2014). It has become an exceptionally important in vivo model for biomedical research, especially to test the toxicity of drugs such as cisplatin or the antibiotics gentamicin (Rocha-Sanchez et al., 2018; Swanhart et al., 2011). Indeed, even at the larval stage, the fish has easily accessible hair cells in its lateral line (a sensory organ under the skin), and a primitive, anatomically simple kidney (Swanhart et al., 2011).

Wertman et al. examined whether 1200 compounds could protect the kidneys and lateral line hair cells of zebrafish larvae against the toxic effects of cisplatin. The screening highlighted 22 molecules, including two that offered the highest levels of protection: dopamine, a compound that nerve cells use to communicate, and L-mimosine, a rare plant non-protein amino acid similar to the amino acid tyrosine (Figure 1). Their protective potential was confirmed in vivo in the primitive kidney and another population of hair cells in zebrafish larvae. In addition, dopamine and L-mimosine did not keep cisplatin from killing cancer cells grown in the laboratory.

**Figure 1.**
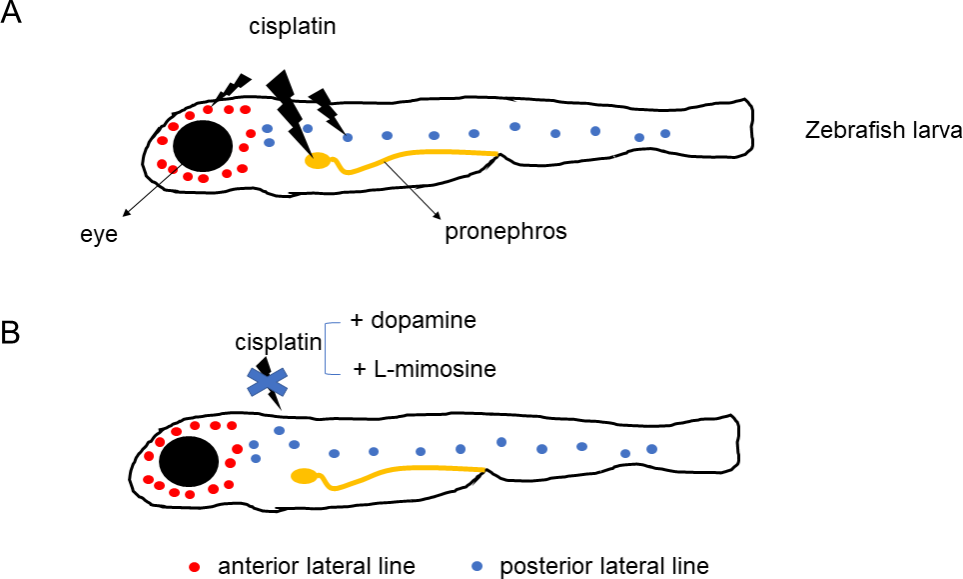
Protective effects of dopamine and L-mimosine against the cancer drug cisplatin. Treatment of zebrafish larvae with cisplatin (panel **A**) impairs the function (lightning bolt icon) of the fish's primitive kidney (pronephros), and of its anterior and posterior lateral line – the organs that display hair cells similar to the ones found in ears. Application of dopamine or L-mimosine (panel **B**) suppresses the toxic effects of cisplatin.

The next step would be to investigate how dopamine and L-mimosine perform this protective role. Organic cation transporters are a family of proteins that help to carry molecules – including dopamine – into cells. In their absence, cisplatin is less toxic for ears and kidneys (Hucke et al., 2019). It is therefore possible that dopamine and L-mimosine compete with cisplatin for access to the transporters: this would result in fewer cisplatin molecules accessing kidney and ear hair cells, ultimately protecting the organs against the cancer drug.

Finally, it is essential to demonstrate that dopamine and L-mimosine do not impair the anticancer activity of cisplatin in vivo, which could also be done in zebrafish larvae. In addition, this animal model could be useful to study neurotoxicity, another potential side effect of the drug. This would allow scientists to investigate whether the two compounds only protect specific organs, or globally interfere with cisplatin activity.

Confirming that dopamine and L-mimosine preserve the anticancer properties of cisplatin in vivo, together with fully understanding how they shield ears and kidneys from the drug’s toxicity should help to develop protective therapies. Ultimately, this would allow more aggressive cancer chemotherapy to be performed, and improve the quality of life of cancer survivors.
